# Impact of ambient fine particulate matter (PM_2.5_) exposure on the risk of influenza-like-illness: a time-series analysis in Beijing, China

**DOI:** 10.1186/s12940-016-0115-2

**Published:** 2016-02-11

**Authors:** Cindy Feng, Jian Li, Wenjie Sun, Yi Zhang, Quanyi Wang

**Affiliations:** School of Public Health, University of Saskatchewan, Saskatoon, SK S7N 5E5 Canada; Department of Biostatistics and Bioinformatics, School of Public Health and Tropical Medicine, Tulane University, New Orleans, LA 70112 USA; School of Food Science, Guangdong Pharmaceutical University, Zhongshan, 528458 China; School of Public Health and Tropical Medicine, Tulane University, New Orleans, LA 70112 USA; Beijing Center for Disease Prevention and Control (CDC), Beijing, 100013 China

**Keywords:** PM_2.5_, Influenza, Meteorological factor, Spline, Generalized additive model

## Abstract

**Background:**

Air pollution in Beijing, especially PM_2.5_, has received increasing attention in the past years. Although exposure to PM_2.5_ has been linked to many health issues, few studies have quantified the impact of PM_2.5_ on the risk of influenza-like illness (ILI). The aim of our study is to investigate the association between daily PM_2.5_ and ILI risk in Beijing, by means of a generalized additive model.

**Methods:**

Daily PM_2.5_, meteorological factors, and influenza-like illness (ILI) counts during January 1, 2008 to December 31, 2014 were retrieved. An inverse Gaussian generalized additive model with log link function was used to flexibly model the nonlinear relationship between the PM_2.5_ (single- and multiday lagged exposure) and ILI risk, adjusted for the weather conditions, seasonal and year trends. We also assessed if the effect of PM_2.5_ differs during flu season versus non-flu season by including the interaction term between PM_2.5_ and flu season in the model. Furthermore, a stratified analysis by age groups was conducted to investigate how the effect of PM_2.5_ differs across age groups.

**Results:**

Our findings suggested a strong positive relationships between PM_2.5_ and ILI risk at the flu season (October-April) (*p*-value < 0.001), after adjusting for the effects of ambient daily temperature and humidity, month and year; whereas no significant association was identified at the non-flu season (May-September) (*p*-value = 0.174). A short term delayed effect of PM_2.5_ was also identified with 2-day moving average (current day to the previous day) of PM_2.5_ yielding the best predictive power. Furthermore, PM_2.5_ was strongly associated with ILI risk across all age groups (*p*-value < 0.001) at the flu season, but the effect was the most pronounced among adults (age 25–59), followed by young adults (age 15–24), school children (age 5–14) and the elderly (age 60+) and the effect of PM_2.5_ was the least pronounced for children under 5 years of age (age < 5).

**Conclusions:**

Ambient PM_2.5_ concentrations were significantly associated with ILI risk in Beijing at the flu season and the effect of PM_2.5_ differed across age groups, in Beijing, China.

## Background

Air pollution has been well documented as a major public health issue for many areas of the world, as a growing body of epidemiological and clinical evidence has shown that pollutants increase the risks of numerous diseases [1–7]. Airborne particulate matter is a mixture of liquid and solid material of varying size and chemical characteristics, which includes dust, dirt, soot, smoke, and liquid droplets emitted into the air. The sizes of the inhalable particles are limited to be those within aerodynamic diameters of 10 *μm* or less (PM_10_) in aerodynamic diameter. PM_10_ consists of two size fractions, fine and coarse, which have both different physiologic and different source characteristics. The particles mechanically generated from agriculture, mining, road traffic, and related sources are generally larger than 2.5 *μm*, which are usually referred to as coarse mass particles (PM_2.5-10_). In contrast, particles resulting from combustion processes are generally less than 2.5 *μm*, which are defined as fine particles (PM_2.5_).

Toxicological and epidemiological studies suggest that PM_2.5_ are especially harmful [2, 4–6, 8, 9], since smaller particles are more likely to penetrate deeper into the lungs and blood streams unfiltered [10]. Studies have shown exposure to PM_2.5_ is associated with a number of adverse health outcomes ranging from respiratory disease [9, 11, 12] to cardiovascular disease [1, 4, 13]. Elevated fine-particulate concentrations are also the cause of mortality [2, 3, 14, 15]. This is also one of the important reasons for WHO designating all countries to have standards for PM_2.5_. In 2013, a longitudinal study involving 312,944 people in nine European countries revealed that that the lung cancer rate rose 22 % for every increase of 10 *μg*/*m*^3^ in PM_10_. The smaller PM_2.5_ were particularly deadly, with a 36 % increase in lung cancer per 10 *μg*/*m*^3^and the effect of PM_2.5_ was not affected by adjustment for PM_2.5-10_ [9]. Other studies also revealed the similar findings showing that PM_10_ and PM_2.5_ are significantly associated with all cause and cause-specific mortality [2]; whereas no such associations were observed for PM_2.5-10_ [2, 6, 8, 16]. Those studies suggested that the proportion of PM_2.5_ in the PM_10_ composition is more important and might be more strongly related to adverse health effects. Thus, PM_2.5_ pollution has gained increasing attention, especially for those living in metropolitan areas [17].

Beijing, the capital city in China, has been suffered with severe air pollution in the last decade due to rapid industrial expansion and the increased number of automobiles on the road. The number of heavy or more severe pollution days (PM_2.5_ > 75 *μg*/*m*^3^) has been hovering over hundred days annually in Beijing [18, 19]. In China, much attention on air pollution has been focused on PM_10_ [20, 21]. Few studies have devoted to study the PM_2.5_ exposure on health impact, partly because of lack of such information, until recently China has released PM_2.5_ concentrations in major cities to the public [22].

Researchers believe that airborne pollution particles provide “condensation nuclei” to which virus droplets attach; however, the quantitative research on the association between air pollution and influenza is still rare, considering that extreme ambient pollution is a biologically plausible risk factor, and that more intense pollution are imminent this century. Despite a recent study showing that PM_2.5_ was associated with monthly influenza cases [22], there are few studies using daily pollution and influenza data, and no study has been conducted to date investigating the effects of PM_2.5_ on influenza risk by age group. Such research is needed, as influenza epidemics constitute a serious public health problem associated with increased morbidity and mortality, especially in high risk populations, with children, the elderly, and patients with chronic diseases being particularly vulnerable to air pollution [23]. As such, it is important to determine if the effect of PM_2.5_ varies over different age groups. Meteorological factors, in particular temperature and humidity, have also been shown contributing to the risk of influenza infections, such that both low temperature and humidity increase the spread of influenza viruses [24, 25]. However, to our knowledge, no studies have precisely examined the association between PM_2.5_ and influenza by age groups, after controlling the confounding effects of meteorological factors.

In the present study, we provide direct evidence to support the role of ambient fine particulate matter exposure, after adjusting for the effects of weather conditions in the dynamics of influenza and thereby address an emerging question fundamental to the understanding of influenza epidemiology. A generalized additive model was utilized to flexibly model the nonlinear relationship between the daily PM_2.5_ and daily influenza risk in Beijing from year 2008 to 2014, while adjusting for the effects of ambient daily temperature and humidity, status of being week day or weekend/holiday, month and year. We also assessed if the effect of PM_2.5_ differs across various age groups. To explore the delayed impact of PM_2.5_, lag effect of PM_2.5_ was also considered.

## Methods

### Data sources and description

Influenza data consisted of reports of daily number of patients seeking medical attention with influenza-like illness (ILI), defined as the one with body temperature more than 38° Celsius and cough or sore throat, from January 1, 2008 to December 31, 2014 in the capital city of China, Beijing. The data was retrieved from the surveillance system at the Beijing Centre of Disease Control [26]. The influenza surveillance system has been reported elsewhere [27]. In brief, the surveillance is conducted in 150 level two and level three hospitals in Beijing, which consists of hospitals from national, city and district level. The data has a reasonable representativeness given that the sentinel hospitals cover all the 16 districts in Beijing, and the data were from the all outpatients related to respiratory disease treatment. Health care system in each sentinel hospital reports the data to the Beijing Centre of Disease Control every day from online system, and staffs in the district Centre of Disease Controls are responsible for data validation.

Average daily measurements of PM_2.5_ from January 1, 2008 to December 31, 2014 were retrieved from an air quality monitoring site at the US Embassy in Beijing, which is located at the Chaoyang district. The Embassy’s air pollution data was used because it recorded detailed measurements of PM_2.5_ over a long period of time, despite originating from only one location. The data was validated and used by other paper [22]. Temperature and relative humidity were considered as the potential confounders of the association between PM_2.5_ and ILI risk. Daily temperatures and relative humidity, spanning the study period, were obtained through the China Weather Network’s outdoor weather reports. Daily counts of ILI, air pollution levels and weather data were linked by date and analyzed. This study was approved by the Institutional Review Board at Beijing Centre of Disease Control [28].

### Statistical analysis

In epidemiological research, one most frequently used model for modeling counts data is the Poisson regression. A severe limitation of the Poisson model is that the mean and variance of the dependent variable are assumed to be equal, conditional on any covariates [29]. In practice, a very common complication when modeling discrete responses is the presence of overdispersion, when the variance of the response is greater than the mean [30]. It is generally caused by positive correlation between responses or by an excess variation between response counts. If overdispersion is present in a dataset, the standard errors of the estimates could be underestimated (i.e. a variable may appear to be significant predictor when it is in fact not significant) [29]. Negative binomial (NB) regression has been suggested as an alternative to the Poisson, which accounts for overdispersion by adding an additional dispersion (variance) parameter to the Poisson model [31]. However, the negative binomial distribution also imposes some constraints on the mean and variance relationship, whose validation also needs to be seriously assessed. Over the past decades, the family of inverse Gaussian distributions [32, 33] has attracted the attention of many researchers in studying the number of event occurrences for a wide range of field. The inverse Gaussian distribution is particularly useful for dealing with data of considerable skewness [34]. In such cases, the choice is made upon the basis of goodness of fit and upon the ease of working with the distribution. As such, we carefully examined the Poisson, the negative binomial and the inverse Gaussian regression models to identify which model fits the data well and fits the data the best. In fact, all three types of distributions belong to the exponential family in a generalized linear modeling framework [35]; therefore, all the interpretation of the regression coefficients are the same, if the same link function is applied. Here, we used the most commonly used log link function for ease of interpretation [36].

To allow for comparability, all models were adjusted for the same meteorological variables (temperature and humidity) and time variables (year and day of the week). We screened all variables for multi-collinearity. All the three types of models can be written in the following form:

$$ \begin{array}{l} \log \left({\mu}_t\right)={\alpha}_0+ \log \left({n}_t\right)+{f}_1\left({\mathrm{PM}}_{2.5,t-p}\right)I\left({\mathrm{flu}\ \mathrm{season}}_{\mathrm{t}}\right)+{f}_2\left({\mathrm{PM}}_{2.5,t-p}\right)I\left({\mathrm{nonflu}\ \mathrm{season}}_t\right)\\ {}\kern0.5em  + {f}_3\left({\mathrm{t}\mathrm{emperature}}_{\mathrm{t}}\right)+{f}_4\left({\mathrm{humidity}}_{\mathrm{t}}\right)+{f}_5\left({\mathrm{month}}_{\mathrm{t}}\right) + {\displaystyle {\sum}_k{\beta}_kI\left({\mathrm{year}}_{\mathrm{t}}=k\right)+\gamma I\left({\mathrm{week}\ \mathrm{day}}_{\mathrm{t}}\right),}\end{array} $$

where *μ*_*t*_ represents the expected mean number of individuals reporting ILI on day *t* and *α*_0_ denote the intercept. We let *n*_*t*_ denote the population size on day *t*, which is estimated by fitting a sigmoid function to the annual population size of Beijing spanning the study period [37–39], since only annual population of Beijing can be retrieved for this study. We define the ILI incidence rate as the ratio of *μ*_*t*_ relative to *n*_*t*_. Following the standard practice in generalized additive models, *f*_*j*_(*x*), _*j* = 1,…,5_, are the penalized smoothing spline functions for PM_2.5_ at flu season (October-April), non- flu season (May-September), temperature, humidity and month, respectively. That is, *f*_*j*_(*x*) = ∑_*i* = 1_^*q*^*b*_*i*_(*x*)*δ*_*i*_, where *b*_*i*_(*x*), *i* = 1, … *q* are a set of basis functions and *δ*_*i*_ are the corresponding regression coefficients. These basis functions are sections of polynomials that join at a number of knot locations. Common type of basis functions include cubic B-splines or thin-plate splines [36]. The smoothness of the spline functions were automatically estimated using unbiased risk estimation [36]. To explore the delayed impact of PM_2.5_ on ILI risk, we lagged PM_2.5_ by *p* days, denoted by *PM*_2.5,*t* − *p*_ representing the measurement of PM_2.5_ taken at day *p* prior to ILI case report date *t*, *p* = 0, 1, … 5. For example, a lag of 0 days (lag 0) corresponds to the current day PM_2.5_, and a lag of 1 day (lag 1) refers to the previous-day PM_2.5_. We also investigated the effect of accumulated exposures of PM_2.5_ on ILI incidence by taking mean of lag01 (PM_2.5_ averaged over the current day and the previous day), and up to mean lag05. We selected a lag according to the lowest Akaike Information Criterion (AIC) [28]. *I*(*A*) is an indicator variable, such that if A is true then *I*(*A*) = 1 and 0, otherwise; *β*_*k*_ is the regression coefficient for year *k* and *γ* is the regression coefficient for weekday. To investigate if the effect of PM_2.5_ varies with age, stratified analysis at different age groups was also conducted, with age groups being classified as <5, 5–14, 15–24, 25–59, ≥60 years old. The statistical analysis was conducted using the *mgcv* package in R [36]. All statistical tests were two-sided and *P*-values with less than 0.05 were considered statistically significant.

## Results

**Fig. 1:**
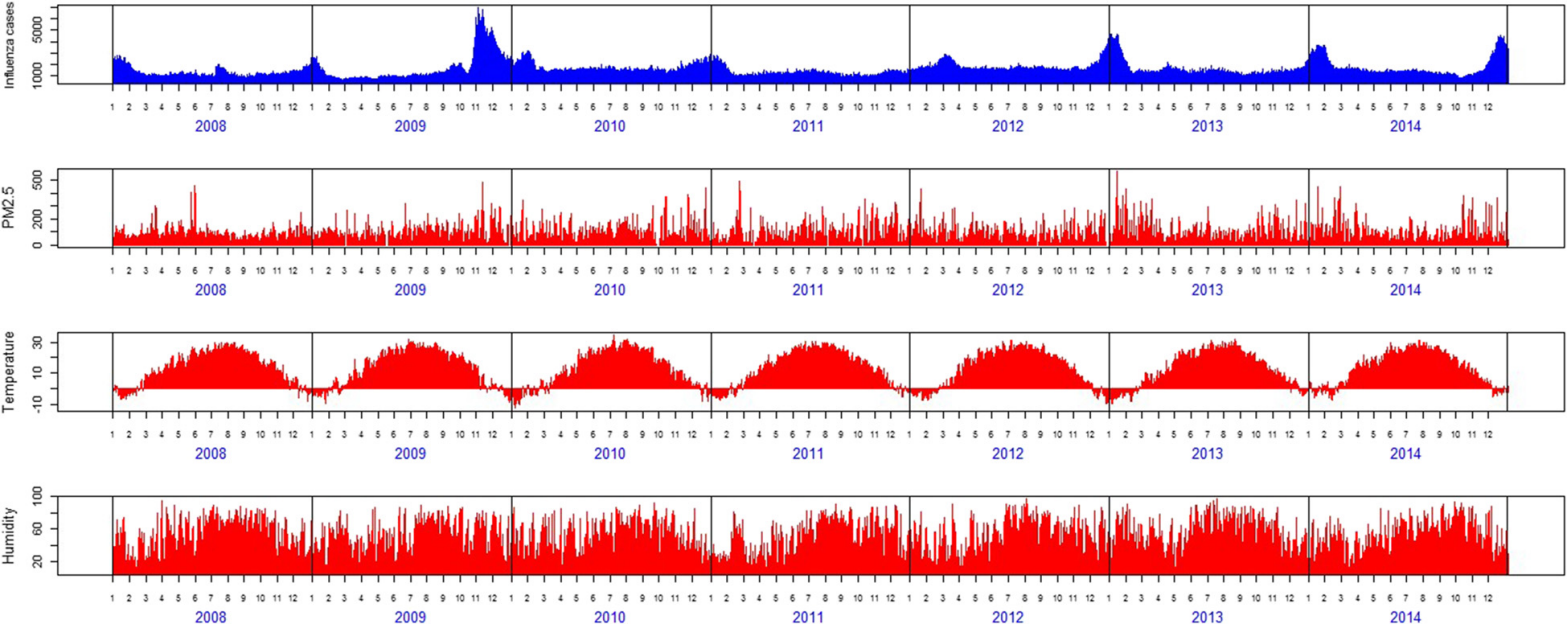
The time course of daily influenza cases, daily PM2.5, daily temperature and average humidity from January 1, 2008 to December 31, 2014

The distributions of daily ILI cases, daily PM_2.5_, daily temporal and humidity from January 1, 2008 to December 31, 2014 are depicted in Fig. 1. On average, there were 1763 ILI cases per day, 695 for age <5, 448 for age 5–14, 191 for age 15–25, 357 for age 25–29 and 71 for age 60+ (Table 1). Daily mean concentration of PM_2.5_ was 100.66 *μg*/*m*^3^, with standard deviation 80.86 *μg*/*m*^3^, which was more than four times higher than the WHO’s guideline of 25 *μg*/*m*^3^. The maximum PM_2.5_ reached at 568.6 *μg*/*m*^3^ on January 12, 2013, which was record breaking [22]. At the flu season (October-April), the number of ILI cases for all age groups, except for <5 years old, tends to be higher than the corresponding the number of ILI cases at the non-flu season (May-September). The median (Q1-Q3) for PM_2.5_ at flu season is 77.62 *μg*/*m*^3^ (44.50 *μg*/*m*^3^–131.00 *μg*/*m*^3^) and at the non-flu season is 83.26 *μg*/*m*^3^ (52.25 *μg*/*m*^3^–115.70 *μg*/*m*^3^). The temperature and humidity tend to be lower at the flu season as compared to the non-flu season with the mean (Q1-Q3) for temperature at flu season is 6.35 °C (-0.90 °C, 14.72 °C) and at the non-flu season is 25.60 °C (23.30 °C, 27.50 °C ); the mean (Q1-Q3) for humidity at flu season is 46 *g*/*m*^3^ (30 *g*/*m*^3^–63 *g*/*m*^3^) and at the non-flu season is 63 *g*/*m*^3^ (48 *g*/*m*^3^–73 *g*/*m*^3^) (Table 1).

**Table 1:** Summary statistics of daily ILI counts, PM_2.5_, and weather conditions in Beijing, China, during January 1, 2008 to December 31, 2014 (Q1, Q2 and Q3 denote the 25^th^, 50^th^ and 75^th^ percentile, respectively)

Variable	Flu season (October-April)						Non-flu season (May-September)					
	Mean ± SD	Minimum	Q1	Q2	Q3	Maximum	Mean ± SD	Minimum	Q1	Q2	Q3	Maximum
*Daily ILI counts by age groups*												
<5	695.45(227.78)	270	529	673	815	1717	657.45(152.24)	357	539	648	782	1088
5–14	448.38(250.29)	124	311	389	505	2794	349.26(86.40)	153	289	341	406	710
15–24	191.29(225.80)	32	90	119	205	2097	104.44(25.47)	44	86	103	120	197
25–59	356.89(294.82)	79	179	246	429	1627	218.73(63.67)	78	176	211	260	512
60+	71.24(53.97)	10	40	54	83	381	51.52(22.86)	16	38	48	62	447
*Fine airborne particulate matter* (*μg*/*m* ^*3*^)												
*PM*_2.5_	100.66(80.86)	2.92	44.50	77.62	131.00	568.00	90.14(53.24)	9.79	52.25	83.26	115.70	463.00
*Meteorological Measures*												
Temperature	7.13(8.87)	−12.50	−0.90	6.35	14.72	26.60	25.09(3.36)	11.20	23.30	25.60	27.50	34.50
Humidity	47.39(19.82)	8	30	46	63	95	59.86(17.63)	13	48	63	73	97

We compared the model fits for the Poisson, negative binomial and inverse Gaussian generalized additive models with log link functions when PM_2.5_ was lagged at single day or moving average ranging from 0 to 5 days prior to ILI reporting date. The detailed model comparison was presented in Table 2. Based on the Akaike’s Information Criterion (AIC) [28], the inverse Gaussian generalized additive model using the 2-day moving average of PM_2.5_ yielded the best fit. We further examined how well do the fitted values of response variable correspond to the observed data using the chi-square goodness of fit test. The results indicated that the inverse Gaussian generalized additive model with the 2-day moving average of PM_2.5_fitted the data very well (deviance = 0.102, df = 2463, *p*-value = 1), where the deviance measures the overall difference between the fitted values and the observed values of the response variable. The inverse Gaussian model provided a much better fit than the negative binomial regression (deviance =2525, df = 2464, *p*-value = 0.193); whereas the Poisson regression yielded a substantially larger deviance and failed to fit the data adequately (deviance = 358761, df = 2448, *p*-value < 0.001). Therefore, the inverse Gaussian generalized additive model was used in the subsequent analyses. All the covariates were statistically significant except for holiday weekend; therefore, it was removed from the subsequent analysis.

**Table 2:** AIC scores for the Poisson, negative binomial and inverse Gaussian generalized additive model with log link function modeling the ILI incidence rate in association with PM_2.5_ interacting with flu season, while adjusting for daily temperature, humidity, month and year effects. The PM_2.5_ is lagged by 0, 1, 2, 3, 4, and 5 days prior to the ILI reporting date and lag01 (PM_2.5_ averaged over the current day and the previous day), lag 02 (PM_2.5_ averaged over the current day, the previous day and 2 days before the current day) and so on, up to mean lag05 (PM_2.5_ averaged over the past 6 days)

lag	Inverse Gaussian	Negative binomial	Poisson
0	36828	37244	381533
1	36842	37249	381169
2	36881	37284	385726
3	36913	37314	390007
4	36919	37320	390605
5	36912	37314	389934
01	**36817**	37237	381769
02	36818	37233	380303
03	36828	37243	381532
04	36840	37251	380915
05	36847	37256	381354

The bolded number indicates the smallest number in the table

**Fig. 2:**
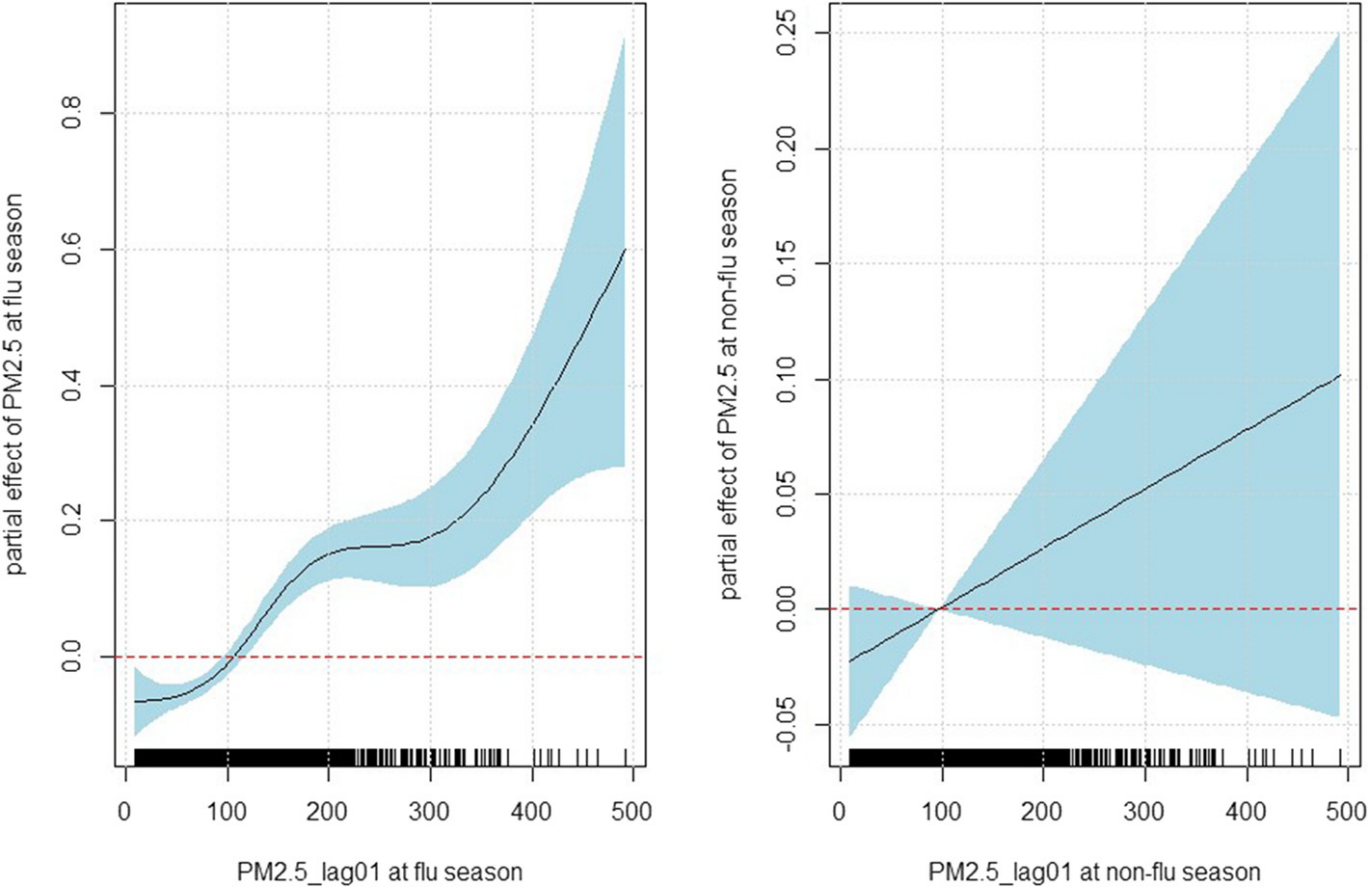
The panels display the estimated partial effect of 2-day moving average (current day to the previous day) of PM_2.5_ at the flu season (October-April) and non-flu season (May-September), based on the inverse Gaussian generalized additive model: $$ \begin{array}{l} \log \left({\mu}_t\right)={\alpha}_0+ \log \left({n}_t\right) + {f}_1\left({\mathrm{PM}}_{2.5, lag01}\right)I\left({\mathrm{flu}\ \mathrm{season}}_{\mathrm{t}}\right)+{f}_2\left({\mathrm{PM}}_{2.5, lag01}\right)I\left({\mathrm{nonflu}\ \mathrm{season}}_t\right)\\ {} + {f}_3\left({\mathrm{t}\mathrm{emperature}}_{\mathrm{t}}\right)+{f}_4\left({\mathrm{humidity}}_{\mathrm{t}}\right)+{f}_5\left({\mathrm{month}}_{\mathrm{t}}\right)+{\displaystyle {\sum}_k{\beta}_kI\left({\mathrm{year}}_{\mathrm{t}}=k\right).}\end{array} $$ The X-axis is the PM_2.5_ concentration (2-day moving average). The solid lines indicate the estimated log relative risk of ILI and the dashed lines indicate the corresponding 95 % confidence intervals

The exposure–response relationships for PM_2.5_ (lag01) and ILI risk at the flu season (left panel of Fig. 2) suggested a very strong positive relationship between PM_2.5_ and ILI risk (*p*-value < 0.001), even after controlling for the weather conditions, seasonal and year trends. The estimated effect (slope) of PM_2.5_ was only marginal when PM_2.5_ was between 0 and about 70 and then increased sharply up until 200 and the trend tended to plateau between 200 and 300, which was followed by a sharp increase afterwards. We also observed a positive relationship between PM_2.5_ and ILI risk at the non-flu season (right panel of Fig. 2), but the effect was not statistically significant (*p*-value = 0.174), since all the pointwise 95 % confidence intervals covered zero and became very wide at higher values of PM_2.5_ because of limited data in this range (Table 1).

**Fig. 3:**
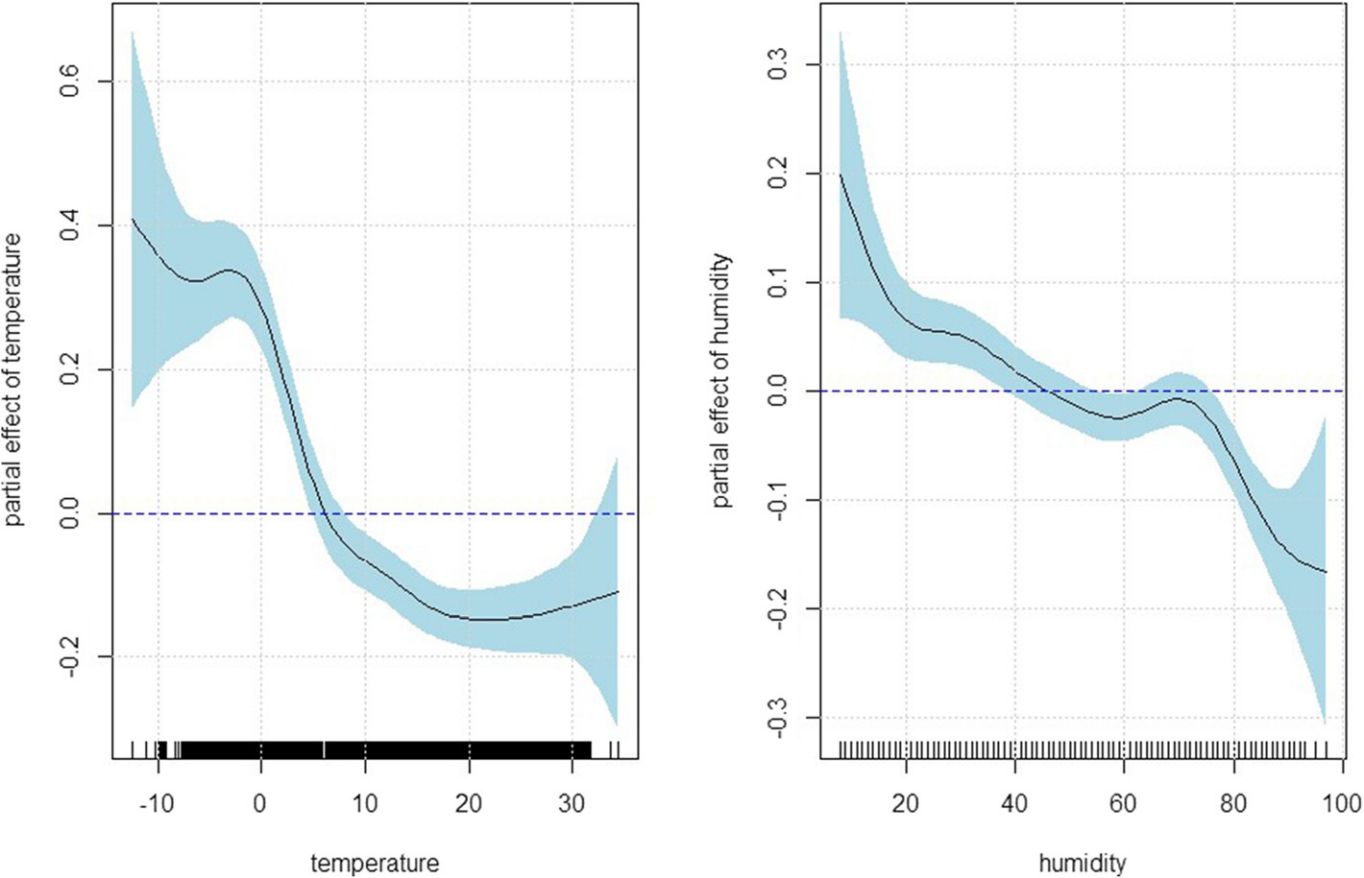
The panels display the estimated partial effect of temperature and humidity based on the inverse Gaussian generalized additive model: $$ \begin{array}{l} \log \left({\mu}_t\right)={\alpha}_0+ \log \left({n}_t\right)+{f}_1\left({\mathrm{PM}}_{2.5, lag01}\right)I\left({\mathrm{flu}\ \mathrm{season}}_{\mathrm{t}}\right)+{f}_2\left({\mathrm{PM}}_{2.5, lag01}\right)I\left({\mathrm{nonflu}\ \mathrm{season}}_t\right)\\ {} + {f}_3\left({\mathrm{t}\mathrm{emperature}}_{\mathrm{t}}\right)+{f}_4\left({\mathrm{humidity}}_{\mathrm{t}}\right)+{f}_5\left({\mathrm{month}}_{\mathrm{t}}\right) + {\displaystyle {\sum}_k{\beta}_kI\left({\mathrm{year}}_{\mathrm{t}}=k\right).}\end{array} $$ The x-axis tick labels in the panels represent the observed values temperature and humidity, respectively. The solid lines indicate the estimated log relative risk of ILI and the dashed lines indicate the corresponding 95 % confidence intervals

The ILI risk stayed high when temperature was below zero and then sharply decreased as temperature increased up until about 20 °C and then plateaued (*p*-value < 0.001), shown in the left panel of Fig. 3. The ILI risk also decreased with increased humidity (*p*-value < 0.001) (right panel of Fig. 3). Such inverse relationship between influenza risk and weather condition is consistent with other studies [40, 41], showing that wintertime cold temperatures increase respiratory morbidity and mortality, which is likely attributed to the fact that the virulence of influenza is expected to be stronger near zero than at subfreezing temperatures and a decrease in temperature makes airways more susceptible to the onset of respiratory infections [41].

**Fig. 4:**
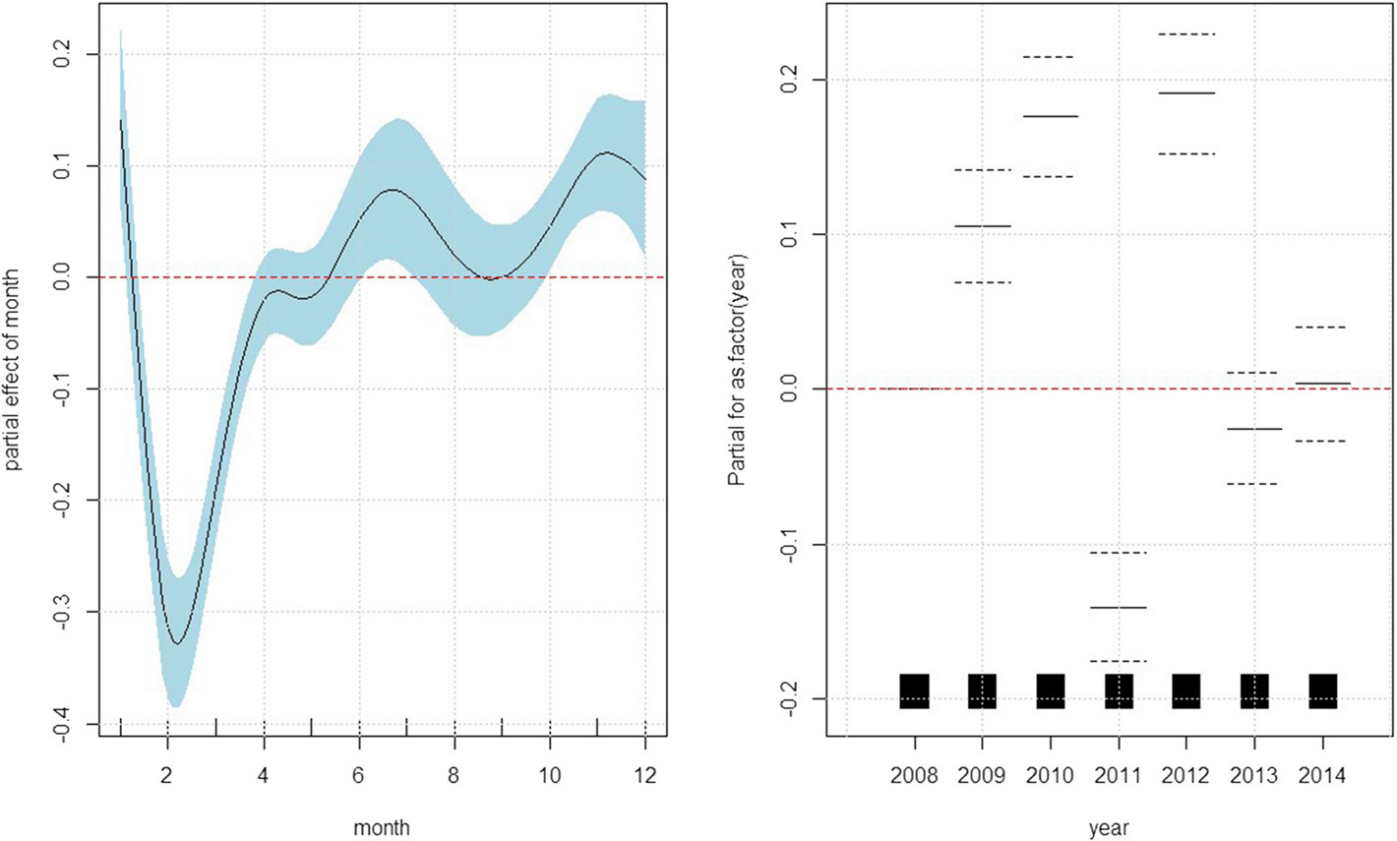
The panels display the estimated partial effect for month and year, based on the inverse Gaussian generalized additive model: $$ \begin{array}{l} \log \left({\mu}_t\right)={\alpha}_0+ \log \left({n}_t\right)+{f}_1\left({\mathrm{PM}}_{2.5, lag01}\right)I\left({\mathrm{flu}\ \mathrm{season}}_{\mathrm{t}}\right)+{f}_2\left({\mathrm{PM}}_{2.5, lag01}\right)I\left({\mathrm{nonflu}\ \mathrm{season}}_t\right)\\ {} + {f}_3\left({\mathrm{t}\mathrm{emperature}}_{\mathrm{t}}\right)+{f}_4\left({\mathrm{humidity}}_{\mathrm{t}}\right)+{f}_5\left({\mathrm{month}}_{\mathrm{t}}\right)+{\displaystyle {\sum}_k{\beta}_kI\left({\mathrm{year}}_{\mathrm{t}}=k\right).}\end{array} $$ The solid lines indicate the estimated log relative risk of ILI and the dashed lines indicate the corresponding 95 % confidence intervals. For the effect of year, year 2008 is set as baseline

The left panel of Fig. 4 indicated that the ILI risk reached the peak during January and then decreased sharply until mid of February followed by a rapid upward trend during early spring and then increased steadily for the rest of the year. The steep drop might be due to large migration out of the city during the Spring festival, which is a traditional festival for family reunion in China. On average, the relative risk of ILI tended to be higher in years 2009, 2011 and 2012 as compared to 2008, 2013 and 2014 and year 2011 had the lowest ILI risk, shown in the right panel of Fig. 4.

**Fig. 5:**
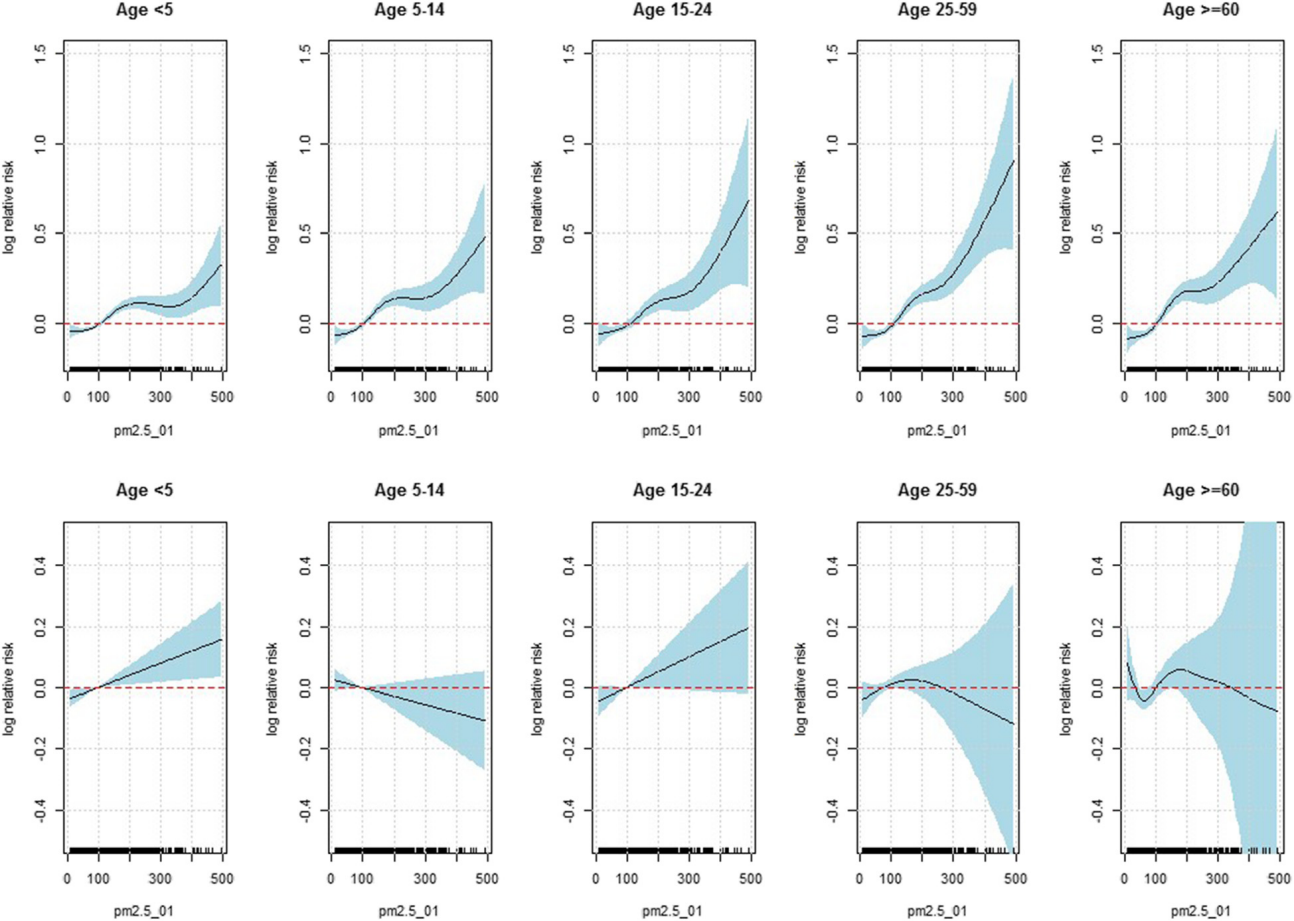
Estimated partial effect of PM_2.5_ based on the stratified analysis for each age group at the flu season (top panels) and non-flu season (bottom panels), based on the inverse Gaussian generalized additive model: $$ \begin{array}{l} \log \left({\mu}_t\right)={\alpha}_0+ \log \left({n}_t\right)+{f}_1\left({\mathrm{PM}}_{2.5, lag01}\right)I\left({\mathrm{flu}\ \mathrm{season}}_{\mathrm{t}}\right) + {f}_2\left({\mathrm{PM}}_{2.5, lag01}\right)I\left({\mathrm{nonflu}\ \mathrm{season}}_t\right)\\ {} + {f}_3\left({\mathrm{t}\mathrm{emperature}}_{\mathrm{t}}\right)+{f}_4\left({\mathrm{humidity}}_{\mathrm{t}}\right)+{f}_5\left({\mathrm{month}}_{\mathrm{t}}\right) + {\displaystyle {\sum}_k{\beta}_kI\left({\mathrm{year}}_{\mathrm{t}}=k\right).}\end{array} $$ The X-axis is the PM_2.5_ concentration (2-day moving average). The solid lines indicate the estimated log relative risk of ILI and the dashed lines indicate the corresponding 95 % confidence intervals

**Fig. 6:**
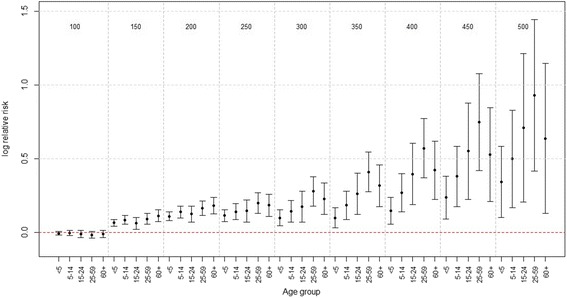
The log relative risk of ILI in association with PM_2.5_ when PM_2.5_ was set as 100 *μg*/*m* ^3^ to 500 *μg*/*m* ^3^ at an increment of 50 *μg*/*m* ^3^, by age groups, when all the other covariates were held at their mean levels

We also investigated the effect of PM_2.5_ by flu season across different age groups. PM_2.5_ had almost no effect on influenza incidence across all age groups at the non-flu season (shown in the bottom panels in Fig. 5), which is consistent with the findings from the overall analysis previously stated. By contrast, at the flu season, the effect (slope) of PM_2.5_ was not significantly different from zero when PM_2.5_ was below about 70 *μg*/*m*^3^; whereas when PM_2.5_ was beyond 70 *μg*/*m*^3^, the effect of PM_2.5_ had an increasing gradient as PM_2.5_ increased. Such pattern was clearly more substantial in the middle aged groups and tended to be most pronounced for age group 25–59 (shown in the top panels of Fig. 5). In general, PM_2.5_ had the greatest effect sizes for adults (age 25–59), followed closely by young adults (age 15–24), and then elderly (age 60+) and school age children (age 5–14) and the PM_2.5_ has the least pronounced effect for the children under 5 years of age (age <5). To further compare the effect of PM_2.5_ across different age groups, Fig. 6 displayed the effect of PM_2.5_ with PM_2.5_ being set as 100 *μg*/*m*^3^ to 500 *μg*/*m*^3^ at an increment of 50 *μg*/*m*^3^. The graph demonstrated that log relative risk of ILI was not substantially different across all age groups when PM_2.5_ was below 250 *μg*/*m*^3^; whereas when PM_2.5_ was around 300 *μg*/*m*^3^, the log relative risk of ILI at age 25–59 remained to be highest compared with other age groups, followed closely by the age groups 15–24 and 60+. The width of the confidence intervals became larger as PM_2.5_ increased, due to the limited observations at the extremely large values of PM_2.5_.

## Discussion

This study is one of the few to investigate the association between PM_2.5_ and ILI. The results of the present study are based on one of the most extensive data sets used thus far in Beijing, China to assess the impact of PM_2.5_ on daily influenza. We provided evidence for the first time, to our knowledge, that PM_2.5_ is non-linearly associated with daily ILI risk based on an inverse Gaussian generalized additive model, after adjusting for the weather conditions, seasonal and year trend. We have assessed the potential lagged effect of PM_2.5_ on ILI risk and our study suggested that PM_2.5_ averaged over the current day and the previous day had the greatest predictive power.

Apart from the statistical evidence of PM exposure enhancing the risk of respiratory viral illness, there has been increasing evidence to support major hypotheses on the biological mechanism underlying this relationship [42–45]. Transmission of viruses via airborne routes is influenced by droplet suspended in the air and the droplet size determines whether the particle will quickly settle to an environmental surface or remain airborne long enough to be inhaled into the respiratory tract of a susceptible host. For example, a study has shown that airborne viruses may be transported by dust storms, which contains many PM [46]. In fact, PM is small enough to suspend in the air for long periods of time, which may provide “condensation nuclei” to which virus droplets attach [45]. Many studies have also reported that PM induces both airway epithelial damage and barrier dysfunction, which could result in a temporary immunosuppressive pulmonary microenvironment [42–45].

Further, the differential effect of PM_2.5_ at the flu season as compared with the non-flu season advances our further understanding of the biological mechanisms of the influenza transmission. Our findings indicated that the ILI risk increased progressively with increased PM_2.5_ at the flu season; whereas the effect of PM_2.5_ was not significant at the non-flu season. We speculate that at the flu season, the amount of viral load was sufficiently high and also the hosts were more susceptible through body cooling and/or drying of the respiratory tract. By contrast, at the non-flu season, even when PM_2.5_ was high, the hosts were not as susceptible to virus infection, might partly because the warmer temperature and higher humidity govern the hosts to be resistant to virus infection leading to the overall lower amount of viral load suspended in the air. As such, both the amount of viral load and pollutant emits as the viral agent could play crucial roles simultaneously determining how PM_2.5_ facilitates the influenza virus transmission.

The other novel contribution of our study is that we assessed the effect of PM_2.5_ on influenza risk by age groups. Our findings were consistent with other studies. For instance, a number of studies have concluded that PM_2.5_ components such as elemental carbon, organic carbon, nitrates, and sulfate were associated with higher proportions of respiratory conditions such as pneumonia, asthma, and bronchitis, based on the hospital admissions for respiratory conditions among children [47–49], in healthy or susceptible and occupational adults [50]. The finding was also consistent with previous studies on elderly populations reporting associations between PM_2.5_levels and hospitalizations for respiratory diseases, including respiratory tract infections, chronic obstructive pulmonary disease, and pneumonia [51, 52]. A number of research have been conducted to identify if there are certain subpopulations are particularly susceptible to PM_10_ and some research has shown that the effect of PM_10_ did not vary with age [53]; however, few studies have been conducted to evaluate if and how the effect of PM_2.5_ differs across different age groups. Our results indicated that at the flu season, PM_2.5_ was strongly associated with ILI risk across all age groups (*p*-value < 0.001), but PM_2.5_ had the greatest effect sizes for adults (age 25–59), followed by young adults (age 15–24), and then elderly (age 60+) and school age children (age 5–14) and the PM_2.5_ has the least pronounced effect for the children under 5 years of age (age <5). The difference in effect sizes across the age groups may be attributable to the exposure differences to the outdoor environments, such as home, schools, workplaces, vehicles, etc. Individuals aged between 25 and 59 or 15–24, who commute to work or school in personal vehicles or public transportation on roadways, are those mostly likely to be exposed to a substantial portion of their daily dose of air pollution during commuting activities [54, 55]. The high exposure to the outdoor pollutants for those people might partially explain why the effect of PM_2.5_ among those people is almost consistently more pronounced compared with other age groups. Interestingly, the number of ILI cases for the groups of individuals between 25 and 59 or 15–24 are markedly lower compared with the under 5 years of age group (Table 1). Similarly, the number of ILI cases for the elderly population is the lowest across all age groups, whereas the impact of PM_2.5_ is almost as high as the young adults and occupational adults. We hypothesize that the elderly people are mostly likely to suffer from chronic diseases, such as asthma, chronic obstructive pulmonary disease (COPD), diabetes, and cardiovascular disease, which may determine their susceptibility to the short-term exposure to elevated levels of air pollution [44, 56]. Such hypothesis is supported by toxicology experiments. For example, researchers have exposed normal and compromised rats to concentrated ambient particles drawn from the outside air in Boston. After three days of exposure to concentrated ambient particles (6 h per day at 228 to 288 μg/m3), mortality was 37 % among rats with induced chronic bronchitis, 19 % among rats with monocrotyline-induced inflammation, and 0 % among normal rats [6]. The elderly with pre-existing chronic respiratory diseases are therefore particularly venerable, since PM can increase oxidative stress, aggravate background inflammation and transient declines in lung function, leading to acute exacerbation of respiratory symptoms [42, 44, 57]. The effect of PM_2.5_ for the under 5 year of age group, albeit weaker, is interesting. The number of ILI cases for the under 5 years of age group is the highest among all the age groups (Table 1), since their lungs are still developing ability to fight off bacterial and viral infections, so they are more susceptible to influenza viruses. Youngsters are therefore urged to stay indoor during flu season to reduce their exposures to influenza viruses. Nevertheless, the effect of PM_2.5_ is still significantly associated with ILI for the children of age under 5, so exposure to outdoor pollutants must occur indoors for the association to be plausible. Ventilation modifies the ability of ambient particles to penetrate indoors. The fraction of the fine particles penetrate indoors as shown to range between 0.3 and 1.0 depending on home ventilation rates [58]. As such, lower exposure to PM_2.5_ for the children who most of the time staying indoors may lead to the reduced effect of PM_2.5_ on ILI incidence.

There are several limitations to our study. First, the PM_2.5_ data was based on only one monitoring site. Although it can reflect the exposure of the PM_2.5_ in Beijing in general, the PM_2.5_ varied from location to location. Future studies with multiple surveillance data are warranted, which could more accurately reflect the exposure. The ILI cases might be also underreported, since not all the patients visit the surveillance hospitals in the network and the people with mild influenza symptoms tend to visit the local physician or stay at home. In addition, misclassification of the influenza may occur, since the ILI symptoms are very similar to other respiratory diseases. Our current study suggested that PM_2.5_, as a specific component of the mixture of air pollutants is significantly associated with ILI. However, different pollution sources can be variably associated with different outcomes interactively and simultaneously and no single pollution source can be attributed to an outcome. A number of studies demonstrated that PM effect remained robust, after controlling the effects of other pollutants, such as the carbon monoxide (CO), sulfur dioxide (SO_2_), Nitrogen dioxide (NO_2_), or ozone (O_3_) [14, 20, 21, 59–62]. A recent systematic review was also conducted based on thirty-three time-series and case-crossover studies in the Chinese population reporting mortality effects of acute exposure to six air quality criteria pollutants including PM_10_, PM_2.5_, SO_2_, NO_2_, O_3_, and CO [63]. Their study suggested per 10 μg/m3 increases in PM_10_ and PM_2.5_, the summary risks of excess death increased 0.32 % (95 % CI: 0.23, 0.40) and 0.51 % (95 % CI: 0.30, 0.73) in respiratory mortality, after controlling for other pollutants. Although most studies consistently suggested the robust association of PM_2.5_ and adverse health outcomes, we recognize other pollutants could be responsible for the adverse health outcomes. Therefore, interpretation our current study should be cautious and replication of the study with more comprehensive exposure data is needed. Furthermore, the effects of PM_2.5_ could be a function of its complex chemical components and composition [64–66]. Therefore, further investigations to advance our understanding of the chemical constituents and sources of PM2.5 are warranted for designing effective emission control policies [67].

In addition, future estimates can be performed for different gender groups, provided that the population size for such groups can be retrieved or estimated. There are other potential sources of heterogeneity, e.g. ones having to do with the geographic distribution of participants and their underlying health conditions. Last, the present study is based on Beijing, and more extensive studies are warranted to ascertain how generalizable our results are to other regions. Given the gap in knowledge, our results provided a good starting point and a priori hypothesis for further studies. Further laboratory studies are in great need to understand the plausible mechanisms underlying the association; as well as longitudinal studies to confirm the causal relationship between exposure to PM_2.5_ and influenza onset.

## Conclusions

Our novel findings suggested that residents in Beijing should be considered at increased risk of ILI during highly polluted days at the flu season (October-April). Such associations are not confounded by long-term time trends, or by weather conditions, all of which were properly controlled in the generalized additive model. While weather is an important predictor of ILI, there was no evidence in these analyses that the PM_2.5_ associations were confounded by weather. Furthermore, the effect of PM_2.5_ was strongly associated with ILI risk across all age groups, but the effect was the most pronounced among adults (age 25–59), followed by young adults (age 15–24), school children (age 5–14) and the elderly (age 60+) and the effect of PM_2.5_ was the least pronounced for the children under 5 years of age (age < 5). These findings provided an improved understanding of interplay of PM_2.5_ and influenza viruses at the flu season and how the effect of PM_2.5_ on ILI risk differed by age groups, which are of great importance in order to enhance the accuracy of surveillance systems, to have more precise predictions on influenza epidemics and pandemics in the future to help both environmental policy-making and public health preparedness.
